# The use of whole genome sequencing to solve an epidemiological puzzle

**DOI:** 10.1002/emmm.201302622

**Published:** 2013-04-02

**Authors:** Alexander Tomasz

**Affiliations:** Laboratory of Microbiology and Infectious Diseases, The Rockefeller UniversityNew York, NY, USA

**Keywords:** bacterial genomes, molecular epidemiology, *Staphylococcus aureus*, whole genome sequencing, zoonotic transmission

With full genome sequencing of bacterial genomes becoming more affordable, the number of publications, which deploy this elegant and modern technique is rapidly increasing. Full genome sequences of hundreds of bacterial strains have been determined and described. However, on reading some of the literature, sometimes one wonders if the use of this method with enormous resolving power is really ‘balanced’ or justified by the specific questions that are being addressed. Or – to put it differently – what exactly were the specific questions for which sequencing was supposed to provide the answer is sometimes unclear.

The paper by Harrison et al. in this issue, poses such specific questions by applying whole genome sequencing (WGS) to resolve an epidemiological puzzle that involves zoonotic transmission of a methicillin-resistant *Staphylococcus aureus* (MRSA) clone in two farms located in Denmark (Harrison et al, 2013).

In both farms the outbreak of MRSA disease involved both the farm animals – cows and dogs in one case and sheep in the other – as well as the farmers. Most interestingly, the MRSA strains involved carried the recently identified and novel *mecC* gene (García-Álvarez et al, 2011) which encodes a homologue of the low affinity penicillin binding protein PBP2A that can provide the bacteria with ‘resistance’ against beta lactam antibiotics particularly cephalosporins (Kim et al, 2012).

The questions addressed in the paper are classical questions of epidemiology. The close geographic vicinity of the two farms and the identical characteristics of the MRSA isolates (clonal complex CC130) which shared identical ST, *spa* type and PFGE profiles suggest that we are observing an outbreak by the same clone at two close geographic locations. Is this the case?

Application of WGS and determination of SNPs in the core genome of the isolates clearly show that this was not the case. Phylogenetic analysis showed that the MRSA isolates could be clearly differentiated into two clusters each specific for the particular farm and isolates recovered from the farmer and livestock from the same farm were virtually identical, differing only by 3–5 SNPs at most.

Was the infection in the farmers preceded by colonization at nasal carriage sites frequently inhabited by MRSA? And did the infection start in the farm animals and passed on to the farmers or the other way around?

In Farm A, nasal swabs and blood isolates showed no SNP differences. This is a potentially important observation since genetic difference between colonizing and invading forms of the same clone has been considered in the literature. The data suggest that colonization of the farmer preceded the blood infection. Isolates from the cows and the farmer only differed by five SNPs indicating clearly a transmission; however, the direction of transmission was not immediately obvious.

In Farm B, the MRSA isolates from sheep 1 and 2 and the farmer only differed by 3–5 SNPs, while the rest of the sheep had multiple (30–40) SNPs. The presence of multiple SNPs suggests either a prolonged circulation of the clone among the animals or multiple introductions of the same MRSA clone into the flock. Together the data suggest that the most likely direction of the zoonotic infection on this farm was from sheep to the human.

In view of the virtually identical MRSA shared by human and animal hosts, the authors also took a close look at the status of virulence genes and the accessory genome in general in the two kinds of isolates, none of which carries the Panton Valentine toxin PVL. This part of the paper also abounds in extremely interesting observations.

This study provides a clear illustration of exactly how and at what point of an epidemiological investigation WGS could increase the resolving power of the study. The authors make the important point that WGS cannot replace other more traditional forms of epidemiological inquiry, but it can greatly and in highly specific ways increase the resolving power of such studies. WGS of isolates from each farm proved beyond doubt the identity and unique nature of the particular MRSA clone recovered from animals and the farmer of the same farm. However, determining the direction of transmission still required a combination of conventional as well as WGS techniques.
